# Inflammatory Foot Dermatoses: The Correlation Among Morphological Patterns, Histopathological Findings, and Patch Test Positivity

**DOI:** 10.7759/cureus.68510

**Published:** 2024-09-03

**Authors:** Muhammed Navas M, Farisa PM, Ali Rishad CM, Gopinath V.P.K

**Affiliations:** 1 Department of Dermatology, Venereology and Leprosy, MES Medical College, Perinthalmanna, IND

**Keywords:** tinea pedis, patch test, plantar psoriasis, lichen planus, acd foot

## Abstract

Objective

In this study, we aimed to investigate the correlation between the clinical diagnosis, histological diagnosis, and patch test positivity among patients with foot dermatoses receiving treatment at the Department of Dermatology, MES Medical College, Perinthalmanna.

Methodology

A hospital-based cross-sectional observational diagnostic evaluation was carried out from January 1, 2019, to December 31, 2019, among 44 patients with foot dermatoses who met the inclusion criteria. A thorough general physical and cutaneous examination was performed to determine the type, extent, and morphology of lesions after obtaining the written informed consent; sociodemographic information and a thorough recording of patient history were gathered. In all cases, a potassium hydroxide (KOH) mount, skin biopsy, and patch testing were performed. Additionally, pus culture and sensitivity tests were conducted in pertinent cases.

Results

Our study found that the most prevalent foot dermatosis, based on clinical diagnosis, was allergic contact dermatitis (ACD) foot, affecting 26 patients (59.0%). This was followed by plantar psoriasis, observed in 13 patients (29.5%), and lichen planus, present in two patients (4.5%). The predominant histological diagnosis was ACD foot in 11 patients (25.0%), followed by plantar psoriasis in 10 patients (22.7%), spongiotic dermatitis in seven patients (15.9%), atopic dermatitis (AD) and psoriasiform dermatitis in four patients (9.1%) each, and tinea pedis in two patients (4.5%). The sensitivity of patch testing in detecting ACD foot was 90.91%, whereas its specificity was 57.58%. The positive and negative predictive values (PPV and NPV) were 41.67% and 95.0% respectively.

Conclusions

Based on our findings, histopathological evaluation is a highly effective diagnostic technique for foot dermatoses, as it demonstrated exceptional diagnostic accuracy (p<0.001). The sensitivity of patch testing in identifying ACD of the foot was 90.91% (p<0.05). This highlighted the usefulness of patch testing as a confirmatory diagnostic tool along with histopathological evaluation for precise diagnosis of ACD of the foot. While laboratory testing can increase diagnostic precision, it cannot replace a clinical examination.

## Introduction

Foot dermatosis is a condition that dermatologists frequently encounter [1]. Various skin conditions such as contact dermatitis, juvenile plantar dermatosis, plantar psoriasis, eczema, and tinea pedis that specifically or mainly affect the feet might be challenging to diagnose without histology. There are subtle clinical distinctions between them that require scrutiny and investigations to be uncovered. Patients with psoriasis have clear, symmetrically spread erythematous plaques, silvery scales, and itchy sores on their fingers, insteps of the foot, and thenar and hypothenar eminences. They also have regular, coarse nail pits without nail-fold lesions. However, poorly defined plaques with asymmetrical distribution and previous exudation indicate dermatitis. There are considerable clinical similarities between the two disorders, and the results of the histology are also often similar.

Several histological traits have been found as psoriasis diagnostic markers, including parakeratosis-containing neutrophilic abscesses, weakening of the granular cell layer, neutrophils in the spinous layer, and tortuous capillaries in the papillary layer of the dermis [2]. Dermatitis is a skin reaction caused by various internal or external factors, such as the physical, chemical, or toxic impacts of different environmental exposures. It is characterized by redness, blistering, and oozing during the acute phase, and by dryness, thickening, scaling, and cracking during the chronic phase [3]. Contact dermatitis is a prevalent dermatological condition that makes up around 4-7% of all consultations with dermatologists [3]. Allergic contact dermatitis (ACD) is a significant ailment, characterized by a hypersensitivity response triggered by specific environmental allergens after previous sensitization [1].

Patch testing is a diagnostic procedure that is employed to identify compounds that can lead to delayed-type allergic reactions in patients. It is particularly useful in identifying allergens that may not be detected through blood tests or skin prick tests. The principle of patch testing involves inducing a localized allergic response in a specific region of the individual's back, wherein the dilute form of the chemical is applied, and to compare with a non-applied or control area on the skin. The patch test kit contains chemicals that are responsible for causing contact allergic eczema in around 85-90% of cases [4].

These chemicals encompass substances found in metals (nickel), rubber, leather, formaldehyde, fragrance, lanolin, hair dye, pharmaceutical medicines, preservatives, and various other chemical additives. It is the gold standard diagnostic evaluation for ACD and a commonly recognized method to identify the triggering allergen. The test is mostly based on the presence of particular T cells that have been stimulated in the allergic reaction and are distributed throughout the body. In vulnerable persons, the absorption of a sufficient amount of the suspect antigens might cause inflammation at the site where it was applied. If the test result is positive, the severity (grade) is evaluated using a grading system [2].

Several studies have established a correlation between clinical characteristics and positive patch-testing findings in foot dermatosis [5]. However, there is a lack of studies that establish a connection between clinical and histological diagnoses and the presence of positive patch test results in foot dermatoses. Therefore, this study aimed to investigate the various morphological and histological traits of foot dermatoses and their correlation with patch testing. This data was compared with the clinical diagnosis, histological diagnosis, and patch test results in individuals with foot dermatoses.

## Materials and methods

Study design and setting

A hospital-based cross-sectional observational diagnostic evaluation was conducted from 1st January 2019 to 31st December 2019, among 44 cases with foot dermatoses who met the eligibility criteria. Patients aged 10 years and above and attending the Department of Dermatology of a tertiary health care institution situated in the northern part of Kerala, India with erythematous/scaly/fissured/oozy lesions exclusively or predominantly involving the foot for more than eight weeks were included in the study. This region has a tropical wet and dry climate. The average temperature during the year of study was approximately 31 ⁰C. The highest temperature recorded in the summer was 42⁰C and the minimum temperature recorded in the year was 24 ⁰C. Patients on long-term steroids, those not willing to participate in the study, patients with contraindications of skin biopsy, and pregnant women were excluded.

Ethical approval and data collection

The Institutional Ethical Committee clearance was obtained (IEC/MES/40/2018). After getting informed written consent, sociodemographic data, and detailed patient history, a complete general physical and cutaneous examination of the lesion's nature, extent, and morphology were recorded. The series used for conducting potassium hydroxide (KOH) mounts, skin biopsies, patch testing, and pus culture and sensitivity tests in all cases is typically known as a diagnostic evaluation series. The pus culture and sensitivity tests were done in relevant cases.

The sample size was derived using n=Z^2^_(1-α/2)_ x P(1-P)/d^2 ^where n=sample size, P=estimated proportion, d=desired precision, and Z^2^_1-α/2_=confidence interval. The expected proportion was 44.7% [6], with an absolute precision of 15 at the 95% confidence level.

n=(1.96)^2^⋅0.447⋅(1−0.447)​/0.15^2^ ≈ 42.2

The total number of samples taken into consideration was n=44. The study included consecutive patients who visited the Dermatology Outpatient Department (OPD) and met the qualifying criteria during the study period.

Statistical analysis

The statistical analysis was conducted using SPSS Statistics software Version 25.0 (IBM Corp., Armonk, NY) for Windows. A Microsoft Excel spreadsheet was used to enter coded data. Descriptive statistics were computed by determining the number of observations and percentages for qualitative variables, and the mean and standard deviation (SD) for quantitative variables. The significance level was set at a p-value of less than 0.05.

## Results

Of the 44 patients, 24 (54.5%) were female, while the proportion of male patients was less than half (45.5%) of the sample. The mean age of the patients was 36.05 ± 19.6 years. Males had a greater mean age of 41.40 ± 21.0 years, while females had a mean age of 31.58 ± 17.7 years. There were more males than females in the age groups of 10-20 and 51-60 years, accounting for 25% of the population in each group. The age group with the highest proportion of females was 10-20 years, constituting 41.7% of the total. The age range of 71-80 years had the fewest number of patients in either gender. The predominant clinical diagnosis among the patients was ACD foot (n=26, 59.1%), followed by plantar psoriasis (n=13, 29.5%), and lichen planus (n=2, 4.5%). One patient (2.3%) each had prurigo nodularis, vasculitis, and lichen simplex chronicus. The KOH test was conducted on all 44 patients, and all of them (100%) tested negative for fungal hyphae.

Among the patients, the most frequent histological diagnosis was ACD foot (n=11, 25%). Plantar psoriasis was diagnosed in 10 patients (22.7%), followed by spongiotic dermatitis in seven patients (15.9%). Atopic dermatitis and psoriasiform dermatitis were each diagnosed in four patients (9.1%). Lichen planus and tinea pedis were observed in two cases, accounting for 4.5% each. One patient (2.3%) each had asteatotic eczema, vasculitis, lichen simplex chronicus, and prurigo nodularis. Out of the 44 patients, 24 (54.5%) had a clinical and histological diagnosis that matched, which was shown to be statistically significant (p<0.001) (Table 1).

**Table 1:** Correlation between clinical and histopathological diagnosis ACD: allergic contact dermatitis; LP: lichen planus; LSC: lichen simplex chronicus

Variables			Histopathological diagnosis, n (%)										
			ACD foot	Spongiotic dermatitis	Atopic dermatitis	Tinea pedis	Plantar psoriasis	Psoriasiform dermatitis	LSC	LP	Asteatotic eczema	Vasculitis	Prurigo nodularis
Clinical diagnosis, n (%)	ACD foot	26 (59.0)	10 (22.7)	7 (15.9)	4 (9.1)	2 (4.5)	1 (2.3)	1 (2.3)	0	0	1 (2.3)	0	0
	Plantar psoriasis	13 (29.5)	1 (2.3)	0	0	0	9 (20.4)	3 (6.8)	0	0	0	0	0
	LP	2 (04.5)	0	0	0	0	0	0	0	2 (4.5)	0	0	0
	LSC	1 (2.3)	0	0	0	0	0	0	1 (2.3)	0	0	0	0
	Prurigo nodularis	1 (2.3)	0	0	0	0	0	0	0	0	0	0	1 (2.3)
	Vasculitis	1 (2.3)	0	0	0	0	0	0	0	0	0	1 (2.3)	0
	Total	44 (100)	11	7	4	2	10	4	1	2	1	1	1

Patch test

A patch test was conducted on all 44 patients, with 24 individuals (54.5%) testing positive. A significant segment of patients (15.9%) exhibited an allergic reaction to the black rubber mix. This was followed by mercaptobenzothiazole, which caused an allergic reaction in 13.6% of patients. Paraphenylenediamine caused an allergic reaction in 9.1% of patients, while potassium dichromate caused an allergic reaction in 6.9% of patients. Two patients each were found to have allergies to epoxy resin and balsam of Peru. In the patch test, 20 individuals (45.5%) showed a negative reaction (Table 2).

**Table 2:** Frequency distribution of allergens as determined by patch test

Allergens	N	%
Black rubber mix	07	15.9
Mercaptobenzothiazole	06	13.6
Paraphenylenediamine	04	09.1
Potassium dichromate	03	6.9
Epoxy resin	02	4.5
Balsam of Peru	02	4.5
Negative	20	45.5

The majority of patients (n=10; 90.9%) had a histopathological diagnosis of ACD foot, which was followed by spongiotic dermatitis in seven patients (100%), atopic dermatitis in four patients (100%), psoriasiform dermatitis in two patients (50%), and plantar psoriasis in one patient (10%). Individuals diagnosed histopathologically with vasculitis, prurigo nodularis, asteatotic eczema, tinea pedis, lichen planus, and lichen simplex chronicus did not exhibit patch test positivity (Table 3).

**Table 3:** Frequency distribution of patch test positivity ACD: allergic contact dermatitis

Variables		Patch test	
Histopathological diagnosis	Total cases	Positive, n (%)	Negative, n (%)
ACD foot	11	10 (90.9)	1 (9.9)
Plantar psoriasis	10	1 (10)	9 (90)
Spongiotic dermatitis	7	7 (100)	0 (0)
Atopic dermatitis	4	4 (100)	0 (0)
Psoriasiform dermatitis	4	2 (50)	2 (50)
Lichen planus	2	0 (0)	2 (100)
Tinea pedis	2	0 (0)	2 (100)
Asteatotic eczema	1	0 (0)	1 (100)
Vasculitis	1	0 (0)	1 (100)
Lichen simplex chronicus	1	0 (0)	1 (100)
Prurigo nodularis	1	0 (0)	1 (100)

ACD foot: clinical diagnosis prediction

The histopathological diagnosis was considered the definitive standard for investigation. The clinical diagnosis predictability of detecting ACD foot demonstrated a sensitivity of 90.91%, indicating a 90.91% accuracy in predicting ACD foot. The positive and negative predictive values (PPV and NPV) were 38.46% and 94.44% respectively. As shown in Table 4, there was a high statistical significance of prediction (p<0.013).

**Table 4:** Clinical diagnosis of ACD foot vs. histopathology P<0.05: statistically significant ACD: allergic contact dermatitis; FN: false negative; FP: false positive; TN: true negative; TP: true positive

Variables		Histopathology report: ACD foot		Total
		Absent	Present	
Clinical findings: ACD foot	Absent	17 TN	01 FN	18
	Present	16 FP	10 TP	26
Total		33	11	44
Chi-square test: p-value: 0.013				
Test prediction for ACD foot		%	95% confidence interval	
Sensitivity		90.91%	58.72% to 99.77%	
Specificity		51.52%	33.54% to 69.20%	
Positive predictive value		38.46%	29.56% to 48.21%	
Negative predictive value		94.44%	71.82% to 99.13%	

The specificity for detecting non-ACD conditions was 51.52%, indicating that the clinical diagnosis correctly identified about half of the non-ACD cases. The high NPV of 94.44% shows that when the clinical diagnosis predicts a case as non-ACD, it is likely accurate, correlating well with the histopathological findings. However, the PPV of 38.46% is relatively low, indicating that many cases identified clinically as ACD might not be confirmed as such by histopathology. This suggests that while the clinical diagnosis is reliable for ruling out ACD (high NPV), it is less reliable for confirming ACD (low PPV), highlighting the need for histopathological confirmation for accurate diagnosis.

Plantar psoriasis - clinical diagnosis prediction

The clinical diagnosis prediction of plantar psoriasis cases demonstrated a sensitivity of 90%, indicating a 90% accuracy in predicting the presence of plantar psoriasis. Clinical diagnostics have a specificity of 88.24% in correctly identifying patients without plantar psoriasis. The NPV was 96.77%, and the PPV was 69.23%. The statistical significance of prediction was determined to be p<0.001 (Table 5).

**Table 5:** Clinical diagnosis of plantar psoriasis vs. histopathology P<0.05: statistically significant FN: false negative; FP: false positive; TN: true negative; TP: true positive

Variables		Histopathology report: plantar psoriasis		Total
		Absent	Present	
Clinical findings: plantar psoriasis	Absent	30 TN	01 FN	31
	Present	04 FP	09 TP	13
Total		34	10	44
Chi-square test: p-value: <0.001				
Test prediction for plantar psoriasis		%	95% confidence interval	
Sensitivity		90.00%	55.50% to 99.75%	
Specificity		88.24%	72.55% to 96.70%	
Positive predictive value		69.23%	46.69% to 85.25%	
Negative predictive value		96.77%	82.31% to 99.49%	

Out of the total of 44 patients, 26 were diagnosed with ACD foot based on clinical evaluation. Among them, (80.7%) exhibited positive results in the patch test. The patch test demonstrated a sensitivity of 80.76%, indicating that it accurately predicts allergic conditions in 80.7% of clinically diagnosed cases of ACD foot. It demonstrated a specificity of 83.77% in accurately diagnosing non-allergic foot problems. The PPV was 80.70%, and the NPV was 41.67%. As presented in Table 6, the prediction yielded a statistically significant result (p=0.036).

**Table 6:** Patch test vs. clinical diagnosis P<0.05: statistically significant ACD: allergic contact dermatitis; FN: false negative; FP: false positive; TN: true negative; TP: true positive

Variables		Clinical diagnosis		Total
		Non-ACD	ACD	
Patch test: ACD foot findings	Negative	15 TN	5 FN	20
	Positive	3 FP	21 TP	24
Total		18	26	44
Chi-square test: p-value: 0.036				
Test prediction for the patch test		%	95% confidence interval	
Sensitivity		80.76%	58.72% to 99.77%	
Specificity		83.34%	39.22% to 74.52%	
Positive predictive value		80.70%	31.52% to 52.57%	
Negative predictive value		41.67%	74.13% to 99.21%	

Patch test vs. histopathological diagnosis

Histopathology was used as the definitive method for analysis. Histopathology identified 33 cases as non-ACD and 11 cases as ACD. Among the patch test results, 20 cases were negative for ACD (19 true negatives and one false negative), while 24 cases were positive (14 false positives and 10 true positives). The patch test demonstrated a sensitivity of 90.90%, indicating a 90.91% accuracy in predicting allergic symptoms. It demonstrated a specificity of 57.58% in accurately diagnosing non-allergic foot diseases. The PPV was 41.67% and the NPV was 95%, suggesting that a negative test result is highly indicative of the absence of ACD. The statistical analysis disclosed that the prediction made was statistically significant (p=0.036). The histopathology test demonstrated a sensitivity of 100%, indicating perfect accuracy in predicting ACD (Table 7).

**Table 7:** Patch test vs. histopathology P<0.05: statistically significant ACD: allergic contact dermatitis; FN: false negative; FP: false positive; TN: true negative; TP: true positive

Variables		Histopathology		Total
		Non-ACD	ACD	
Patch test: ACD foot findings	Negative	19 TN	1 FN	20
	Positive	14 FP	10 TP	24
Total		33	11	44
Chi-square test: p-value: 0.036				
Test prediction for the patch test		%	95% confidence interval	
Sensitivity		90.91%	58.72% to 99.77%	
Specificity		57.58%	39.22% to 74.52%	
Positive predictive value		41.67%	31.52% to 52.57%	
Negative predictive value		95.00%	74.13% to 99.21%	

The patch test and histopathology both correlate with clinical outcomes, each providing valuable insights into the diagnosis of ACD foot. The combination of both tests could enhance the overall diagnostic accuracy, reducing the risk of misdiagnosis when clinical evaluations alone are inconclusive.

## Discussion

Diagnosing inflammatory skin conditions that specifically affect the foot can be extremely challenging due to their strong resemblance to one another. However, an accurate diagnosis is crucial due to the large variations in treatment choices for different foot dermatoses. Among the 44 patients in our investigation, 24 were females, accounting for 54.5% of the overall population. Males constituted less than half of the entire population (n=20, 45.5%), resulting in a female-to-male ratio of 1.2:1, which aligns with a similar prior study [7]. In contrast, Kang et al. reported a slight male predominance, with a female-to-male ratio of 1:1.01 [8].

The mean age of patients in the current study was 36.05 ± 19.6 years. Males exhibited a higher prevalence of ACD in the age groups of 10-20 and 51-60 years, encompassing 25% of the population in both age ranges. Erythema was observed in 27 individuals, accounting for 61.36% of the cases. Priya et al. [9] have also made similar findings. However, Suryanarayan et al. [10] documented that the most commonly observed symptoms were papules and vesicles. Foot dermatitis primarily affects the foot, with minimal involvement of other areas of the body. In our study, the most frequently affected area was the dorsum of the foot (57.8%), which corresponds to the shape of the footwear. Priya et al. [9] have also reported similar findings.

We diagnosed ACD foot in 26 out of 44 patients (59%), plantar psoriasis in 13 individuals (29.5%), lichen planus in two patients (4.5%), and lichen simplex chronicus, prurigo nodularis, and vasculitis in one patient each (2.3%). Kang et al. examined the epidemiological characteristics of palmoplantar dermatoses in a group of 237 patients. The most prevalent dermatoses were palmoplantar pustulosis (23.2%), verruca (11.4%), pompholyx (10.1%), palmoplantar keratoderma (8.9%), and contact dermatitis (8.0%) [8]. Hongal et al. [7] observed that the most prevalent palmoplantar dermatoses were palmoplantar psoriasis (20.7%), moniliasis (19%), palmoplantar hyperhidrosis (7%), keratolysis exfoliativa (6%), and pitted keratolysis (6%).

Our study included 11 individuals who were diagnosed with ACD foot based on histological examination (representing 25% of the whole sample). Among these, 10 (90.1%) were also clinically diagnosed with ACD foot, whereas one patient (9.9%) was diagnosed with plantar psoriasis. Out of the 26 patients diagnosed with ACD foot, 59% were confirmed to have ACD foot after histological examination. Specifically, 10 patients (38.6%) had ACD foot, seven patients (26.9%) had spongiotic dermatitis, four patients (15.38%) had atopic dermatitis, and one patient each (3.8%) had plantar psoriasis, psoriasiform dermatitis, lichen simplex chronicus, and asteatotic eczema. Histological analysis revealed that two individuals (4.5%) who were clinically diagnosed with ACD foot really had tinea pedis.

Among the 13 patients initially diagnosed with plantar psoriasis, histopathology confirmed the initial diagnosis in nine patients (69.2%). However, three patients (23.7%) were found to have psoriasiform dermatitis, a condition that can mimic psoriasis both clinically and histologically but has distinct etiologies, often related to inflammatory or allergic processes rather than the autoimmune mechanisms typically associated with psoriasis, and one patient (7.6%) had ACD foot following histopathology. ACD of the foot results from contact with various allergens, such as rubber or leather in footwear, and its clinical presentation can closely resemble that of psoriasis, including erythematous and scaly plaques.

The initial clinical diagnosis of plantar psoriasis can sometimes be challenging due to its similar presentation to other dermatological conditions. Histopathological examination is often essential to confirm the diagnosis and distinguish it from conditions with overlapping clinical features. This differentiation is crucial, as the management strategies for each condition differ significantly. Consistent with the study by Lallas et al. [11], these results highlight the risk of misdiagnosis from clinical examination alone, underscoring the need for histological analysis to ensure accurate diagnosis and treatment. Chougule et al. [12] found that the most prevalent kind of dermatitis was lichen simplex chronicus, accounting for 36% of cases, closely followed by discoid eczema at 18.5%, ACD at 14.5%, and stasis eczema at 7.5%. Rao et al. examined the histology of palmoplantar psoriasis and hyperkeratotic palmoplantar dermatitis and determined that these two disorders share some similar histopathological characteristics. Only a limited number of characteristics were identified as highly indicative of psoriasis in this investigation [13].

Our analysis revealed that 24 patients (54.5%) tested positive for patch tests. Aithal et al. found that out of 50 patients, 30 (60%) tested positive in patch tests [14]. The predominant allergens identified in this investigation were black rubber mix (15.9%), mercaptobenzothiazole (13.6%), paraphenylenediamine (9.1%), and potassium dichromate (6.9%). Epoxy resin and balsam of Peru were each found in 4.5% of cases. Chowdary et al. [15] and Bajaj et al. [6] identified potassium dichromate as the most prevalent allergen for foot eczema. However, Epan et al. [16] found that mercaptobenzothiazole was a common sensitizer, ranking second in our sample. ACD foot is primarily caused by chemicals used in metal buckles, leather processing, dyes in shoes and socks, adhesives, plastic or rubber shoes, and polishing components. According to Bajaj et al., out of 105 patients with foot eczema, 47 exhibited a positive reaction to either one or more than one allergens found in shoes [6].

Our study revealed that 90.1% (10/11) of patients diagnosed with ACD demonstrated positive patch test results, indicating a sensitivity of 90.91%. This suggests that the test accurately predicts ACD foot in 90.91% of cases. The patch test had a specificity of 57.58% to determine non-ACD disorders. The PPV was 41.67%, indicating a low probability of a positive result being accurate. The NPV was 95.0%, indicating a high probability of a negative result being accurate. Nethercott et al. [17] and Diepgen et al. [18] documented that the patch test had a sensitivity and specificity of roughly 70% to 90%, which correlates with the histological findings indicating ACD foot. Hence, it is advisable to perform skin biopsy and patch testing in individuals suspected to have footwear dermatitis to enhance the precision of diagnosis while maximizing the effectiveness of treatment.

This study has a few limitations that should be acknowledged. These include a small sample size limiting generalizability, a cross-sectional design preventing the scope of longitudinal insights, potential selection bias due to data gathered from only a single center, and the exclusion of newer diagnostic technologies. Additionally, while patch testing showed high sensitivity, its moderate specificity suggests the need to exercise caution in clinical interpretation. Further research is needed to enhance diagnostic precision and expand our understanding of these conditions.

## Conclusions

Confirmatory diagnosis in the majority of foot dermatitis cases often requires laboratory assessment, which may include procedures such as patch testing, skin biopsy, and KOH test. Our findings show that histopathological evaluation is a highly useful diagnostic tool for foot dermatoses, demonstrating a high level of diagnostic accuracy (p <0.001). The sensitivity of patch testing in identifying ACD foot was 90.91%, making it also a statistically significant diagnostic tool. This emphasizes the necessity of histopathology and patch testing in detecting foot ACD. Although laboratory assessment cannot replace clinical examination, it can enhance diagnostic precision, leading to improved treatment outcomes. Accurate diagnosis not only facilitates targeted therapy but also enhances patient outcomes by minimizing the risk of mismanagement and promoting effective symptom relief. As such, a thorough understanding of the distinctive features and diagnostic criteria of foot dermatoses is essential for clinicians to navigate these complexities and provide optimal care.
